# Disseminated intravascular coagulation or extended intravascular coagulation in massive pulmonary embolism

**DOI:** 10.1111/j.1538-7836.2010.03891.x

**Published:** 2010-07

**Authors:** M LEVI

**Affiliations:** Department of Vascular Medicine and Internal Medicine, Academic Medical Center, University of Amsterdamthe Netherlands

## Abstract

*See also* Leitner JM, Jilma B, Spiel AO, Sterz F, Laggner AN, Janata KM. Massive pulmonary embolism leading to cardiac arrest is associated with consumptive coagulopathy presenting as disseminated intravascular coagulation. This issue, pp 1477–82; Thachil J. DIC score predicts mortality in massive clot coagulopathy as a result of extensive pulmonary embolism: a rebuttal. This issue, pp 1657–8; Leitner JM, Janata-Schwatzek K, Spiel AO, Sterz F, Laggner AN, Jilma B. DIC score predicts mortality in massive clot coagulopathy as a result of extensive pulmonary embolism: reply to a rebuttal. This issue, pp 1658–9.

Systemic activation of coagulation may occur in the context of a variety of clinical situations. This coagulation activation may only be detectable by measuring sensitive molecular markers for activation of coagulation factors and pathways, but can become clinically manifest as changes in routine coagulation tests may occur [1]. The spectrum of clinically manifest coagulation activation ranges from a small decrease in platelet count and sub-clinical prolongation of global clotting times to its most extreme form, for example fulminant disseminated intravascular coagulation (DIC), characterized by gross laboratory abnormalities and simultaneous widespread microvascular thrombosis and profuse bleeding from various sites [2,3]. DIC is not a disease or a symptom but a syndrome, which is always secondary to an underlying disorder. Although these conditions may be very different, the pathogenesis of DIC seems to follow similar lines. Briefly, intravascular fibrin deposition is a result of tissue factor-mediated thrombin generation that is insufficiently balanced by dysfunctional physiologic anticoagulant mechanisms, such as the antithrombin system and the protein C system [4]. In addition to enhanced fibrin formation, fibrin removal is impaired as a result of depression of the fibrinolytic system caused by high circulating levels of the fibrinolytic inhibitor PAI-1.

The traditional list of underlying diseases known to be associated with DIC includes infectious diseases, systemic inflammatory conditions, trauma and cancer, obstetrical complications and severe toxical and immunological reactions [3]. In this issue of the *Journal of Thrombosis and Haemostasis*, Leitner *et al.* [5] propose to add a new diagnosis to that list. They report on a series of 113 patients with massive pulmonary embolism, serious enough to require cardiopulmonary resuscitation. These patients had a prolonged prothrombin time, lower platelet count and reduced antithrombin levels as compared with subjects with pulmonary embolism that did not need cardiopulmonary resuscitation. Nine percent of the patients that needed resuscitation had DIC, according to the criteria of the International Society of Thrombosis and Hemostasis [6]. Based on these findings, the authors conclude that massive pulmonary embolism could be an eliciting factor for DIC. While this could indeed be the case, an alternative explanation might also be proposed: cardiopulmonary collapse and resuscitation as a consequence of the embolus rather than the pulmonary embolism itself could be responsible for the activation of coagulation. Indeed, several studies have indicated that patients with (out of hospital) cardiac arrest display signs of intravascular coagulation activation and subsequent fibrin deposition [7]. The mechanism underlying this widespread coagulation activation may be a systemic inflammatory response as a result of ischemia reperfusion in patients with circulatory arrest and/or hypoxia [8]. Studies in patients during and after cardiopulmonary arrest have shown enhanced tissue factor expression in combination with reduced tissue factor pathway inhibitor [9]. In addition, this activation of coagulation and fibrin formation is insufficiently balanced by endogenous fibrin removal, as a result of high levels of PAI-1 [10,11]. Indeed, both mechanisms are crucial in the pathogenesis of DIC. Contradictory to the hypothesis that the resuscitation *per se* is responsible for the systemic coagulation activation Leitner *et al.* has shown that patients that were resuscitated for reasons other than pulmonary embolism (i.e. resuscitation as a result of primary cardiac causes) had a markedly less intense activation of coagulation than patients with pulmonary embolism. Hence, it is likely that a pulmonary clot itself may contribute to the coagulopathy. Following that conclusion, it may be suggested that the presence of a large intravascular thrombus in the pulmonary circulation may result in insufficiently contained thrombin generation at the clot surface and overflow of active coagulation factors into the systemic circulation. In that case, extended intravascular coagulation may be a better term than disseminated intravascular coagulation. Similarly, large aortic aneurysms with clots in the false lumen or large arteriovascular malformations have been associated with a consumption coagulopathy mimicking DIC [12–15].

Another interesting finding of Leitner *et al.* is the predictive value of the DIC score for mortality in patients with pulmonary embolism. This is in complete agreement with the prognostic utility of the DIC score in many other clinical situations [16,17]. Surprisingly, there was also a strong correlation between the DIC score and in-hospital mortality in patients with pulmonary embolism who did not require resuscitation. This may be a useful observation as this scoring system may be used to select patients that need more intensive monitoring and treatment compared with the standard management of pulmonary embolism. For example, the use of thrombolytic treatment, in most institutions reserved for patients who present with pulmonary embolism complicated by severe cardiopulmonary insufficiency, may result in a better outcome in these high-risk patients. Obviously, the efficacy of such an approach definitively needs prospective validation in a sound clinical trial, which should also address the safety of more intense antithrombotic strategies in patients with an overt coagulopathy.

In conclusion, based on the present findings massive pulmonary embolism in combination with cardiopulmonary resuscitation can be associated with DIC. The presence of DIC seems to be related to an adverse outcome after pulmonary embolism, also in patients that do not need resuscitation. These findings may be helpful in developing more tailored management strategies in patients that present with pulmonary embolism.
